# GPT-4o is more like a real person: potentials in surgical oncology

**DOI:** 10.1097/JS9.0000000000001898

**Published:** 2024-07-02

**Authors:** Zaijie Sun, Jia Yang, Nan Zhang, Haiyang Wu, Cheng Li

**Affiliations:** a Department of Orthopaedics, Xiangyang Central Hospital, Affiliated Hospital of Hubei University of Arts and Science, Xiangyang; b Department of Orthopedics, Jincheng General Hospital, Jincheng, Shanxi Province; c Department of Gynecological Tumor Ward, The Third Affiliated Hospital of Zhengzhou University; d Department of Orthopaedics, The First Affiliated Hospital of Zhengzhou University, Zhengzhou; e Department of Spine Surgery, Wangjing Hospital, China Academy of Chinese Medical Sciences, Beijing, People’s Republic of China; f Center for Musculoskeletal Surgery (CMSC), Charité-Universitätsmedizin Berlin, Corporate Member of Freie Universität Berlin, Humboldt University of Berlin, Berlin Institute of Health, Berlin, Germany

*Dear Editor,*

In today’s rapidly advancing field of modern medicine, Generative Artificial Intelligence (GAI) is increasingly becoming a vital tool across various medical domains due to its powerful data processing and analytical capabilities. The potential applications of GAI are particularly promising in the complex and challenging branch of oncological surgery. Oncological surgery encompasses the diagnosis, surgical treatment, and postoperative management of cancer, each step requiring precise and efficient decision support. However, traditional diagnostic and therapeutic methods often rely on oncological surgeons’ experience and limited medical records, which, to some extent, constrains the quality and efficiency of healthcare services. GAI, especially natural language processing models such as ChatGPT, can provide significant support in interpreting medical data, patient consultation, and surgical planning through large-scale data training and deep learning techniques. ChatGPT is capable of processing vast amounts of medical literature and clinical data, generating treatment recommendations that adhere to medical standards, and assisting physicians in making more accurate decisions. Numerous clinical studies have already evaluated the application of ChatGPT versions GPT-3.5 and GPT-4 in oncological surgery^1,2^. However, the GPT-4o version, released just a month ago, has garnered widespread attention for its superior performance^3,4^. In this study, we will explore several applications of GPT-4o in surgical oncology by highlighting its advancements with the comparison of previous models such as ChatGPT-3.5 and ChatGPT-4.

In the context of oncological surgery, GPT-4o’s real-time video dialogue and emotion recognition capabilities hold significant potential and practical value. For instance, during preoperative consultations, patients often experience nervousness and anxiety, which can affect their emotions as well as their understanding and acceptance of surgical and treatment plans. GPT-4o facilitates real-time communication with patients and employs its emotion recognition feature to detect their emotional states. During the GPT-4o launch event, the host expressed feeling somewhat nervous and began to breathe rapidly. GPT-4o detected the change in his breathing, sensed his anxiety, and provided guidance to calm his nerves. This simple scenario highlights the strength of GPT-4o’s real-time video dialogue and emotion recognition capabilities. In future clinical care settings, GPT-4o could function as a virtual assistant, offering personalized and empathetic explanations and responses by identifying patients’ emotional states. For example, when a patient exhibits anxiety during a preoperative consultation, GPT-4o is able to provide immediate reassurance, explain the surgical process and expected outcomes, and help alleviate the patient’s anxiety. Additionally, by monitoring patients’ emotional changes, GPT-4o can promptly inform physicians, enabling them to better understand the patient’s psychological state and adjust their communication strategies accordingly.

In addition, one of the most significant advancements of GPT-4o over its predecessors is its exceptional multimodal processing capability. Unlike ChatGPT-3.5 and ChatGPT-4, which primarily focus on text input, GPT-4o seamlessly integrates text, audio, and image data^5^. This multimodal ability enables GPT-4o to achieve broader and deeper applications across various fields, with particularly notable performance in precision medicine. In this domain, GPT-4o combines genomic data with clinical and imaging data to offer unprecedented diagnostic and treatment solutions. It could analyze genetic mutations alongside clinical history and imaging data, providing a comprehensive diagnostic perspective. For instance, in breast cancer cases, traditional treatments often rely on limited genetic and clinical information. In contrast, GPT-4o comprehensively analyzes the genomic data, hormone receptor status, tumor size, location, and spread. This allows oncologists to develop highly personalized treatment plans based on the unique genetic and molecular characteristics of each tumor, recommending the most effective combinations of surgery, chemotherapy, and targeted therapy. This integrated data analysis capability enables GPT-4o to provide more precise and personalized treatment plans. Additionally, GPT-4o’s multimodal processing could monitor patients’ responses in real time during treatment, dynamically adjusting treatment plans through regular analysis of new imaging data and clinical reports. This real-time feedback mechanism enhances treatment precision and significantly reduces side effects.

Moreover, GPT-4o has achieved significant advancements in handling non-English languages, establishing itself as a valuable tool in diverse and multilingual healthcare environments. Oncological surgery typically involves complex medical terminology and detailed medical records, where precise communication is crucial for ensuring high-quality care. However, in practice, patients and healthcare providers may not share a common language, especially in multiethnic and multilingual regions. This is where GPT-4’s multilingual processing capabilities become particularly important. GPT-4o can efficiently translate medical documents, including medical records, surgical reports, and treatment plans. Accurate translation of these documents is vital for the treatment of cancer patients. Through GPT-4o, medical institutions could ensure accurate translation between different languages, preventing misunderstandings or errors due to language barriers, thereby ensuring patients receive the correct treatment. During medical consultations, if there is a language barrier between the patient and the doctor, communication efficiency and accuracy are compromised. With GPT-4o’s real-time interpretation capabilities, doctors and patients can communicate seamlessly, allowing patients to accurately describe their conditions and doctors to promptly provide professional advice and treatment plans. Additionally, GPT-4o’s robust language processing capabilities have brought revolutionary changes to academic exchanges in the field of oncological surgery. International oncological surgery conferences and publications are typically conducted in English, posing a language barrier for researchers from non-English-speaking countries. GPT-4o may assist in translating academic papers, making research findings from non-English-speaking countries more accessible and recognized by the international community, thus promoting global academic exchange and collaboration.

In conclusion, GPT-4o offers numerous advancements over previous models like ChatGPT-3.5 and ChatGPT-4, making it a powerful tool in the field of surgical oncology. Here, we provide additional insights into the pivotal role of GPT-4o, enhancing the existing discussion. We believe that the release of GPT-4o will catalyze an increase in clinical studies investigating its applications in the diagnosis and treatment of various cancers^5,6^. However, it is crucial to address ethical considerations and establish comprehensive guidelines to fully leverage the benefits of this groundbreaking technology.

## Ethical approval

This study does not include any individual-level data and thus does not require any ethical approval.

## Source of funding

This study is supported by China Postdoctoral Science Foundation (2022M720385) and Beijing JST Research Funding (YGQ-202313).

## Author contribution

Z.S., J.Y., and N.Z.: conceptualization, methodology, data curation, formal analysis, resources, investigation, and writing – original draft; H.W. and C.L.: conceptualization, methodology, formal analysis, resources, investigation, and writing – review and editing.

## Conflicts of interest disclosure

The authors declare no conflicts of interest.

## Research registration unique identifying number (UIN)

1. Name of the registry: not applicable.
2. Unique identifying number or registration ID: not applicable.
3. Hyperlink to your specific registration (must be publicly accessible and will be checked): not applicable.

## Guarantor

Nan Zhang, Haiyang Wu, and Cheng Li.

## Data availability statement

The data underlying this article will be shared by the corresponding author on reasonable request.
